# Polymyxin B complexation enhances the antimicrobial potential of graphene oxide

**DOI:** 10.3389/fcimb.2023.1209563

**Published:** 2023-06-21

**Authors:** Santosh Pandit, Lucas Jacquemin, Jian Zhang, Zhengfeng Gao, Yuta Nishina, Rikke Louise Meyer, Ivan Mijakovic, Alberto Bianco, Chengfang Pang

**Affiliations:** ^1^Systems and Synthetic Biology Division, Department of Life Sciences, Chalmers University of Technology, Gothenburg, Sweden; ^2^CNRS, Immunology, Immunopathology and Therapeutic Chemistry, UPR 3572, University of Strasbourg, ISIS, Strasbourg, France; ^3^Research Core for Interdisciplinary Sciences, Okayama University, Okayama, Japan; ^4^Graduate School of Natural Science and Technology, Okayama University, Okayama, Japan; ^5^Interdisciplinary Nanoscience Center, Aarhus University, Aarhus, Denmark; ^6^Department of Biology, Aarhus University, Aarhus, Denmark; ^7^The Novo Nordisk Foundation, Center for Biosustainability, Technical University of Denmark, Kongens Lyngby, Denmark; ^8^Research Group for Genomic Epidemiology, National Food Institute, Technical University of Denmark, Kongens Lyngby, Denmark; ^9^The Intelligent Drug Delivery and Sensing Using Microcontainers and Nanomechanics, Department of Health Technology, Technical University of Denmark, Kongens Lyngby, Denmark

**Keywords:** carbon materials, antibiotics, bacteria, adhesion, biofilm, antimicrobial peptide

## Abstract

**Introduction:**

The antibacterial activity of graphene oxide (GO) has been widely explored and tested against various pathogenic bacterial strains. Although antimicrobial activity of GO against planktonic bacterial cells was demonstrated, its bacteriostatic and bactericidal effect alone is not sufficient to damage sedentary and well protected bacterial cells inside biofilms. Thus, to be utilized as an effective antibacterial agent, it is necessary to improve the antibacterial activity of GO either by integration with other nanomaterials or by attachment of antimicrobial agents. In this study, antimicrobial peptide polymyxin B (PMB) was adsorbed onto the surface of pristine GO and GO functionalized with triethylene glycol.

**Methods:**

The antibacterial effects of the resulting materials were examined by evaluating minimum inhibitory concentration, minimum bactericidal concentration, time kill assay, live/dead viability staining and scanning electron microscopy.

**Results and discussion:**

PMB adsorption significantly enhanced the bacteriostatic and bactericidal activity of GO against both planktonic cells and bacterial cells in biofilms. Furthermore, the coatings of PMB-adsorbed GO applied to catheter tubes strongly mitigated biofilm formation, by preventing bacterial adhesion and killing the bacterial cells that managed to attach. The presented results suggest that antibacterial peptide absorption can significantly enhance the antibacterial activity of GO and the resulting material can be effectively used not only against planktonic bacteria but also against infectious biofilms.

## 1 Introduction

Bacteria grow either as solitary cells in planktonic form or as multicellular structures known as biofilms. In comparison to planktonic cells, life in a biofilm increases bacterial survival in adverse conditions and promotes pathogenicity as protects bacteria from the immune system (Donlan and Costerton, 2002; Helgadóttir et al., 2017; Sharma et al., 2019). In a world where antibiotic-resistant harmful bacteria are proliferating at an alarming rate, finding solutions to counter the infections has become a major public health concern (Aslam et al., 2018). Unfortunately, very little has been achieved in combating this problem through the discovery of new antibiotics (Miethke et al., 2021).

Graphene oxide (GO) has been reported for its antimicrobial activity against a range of Gram-positive and Gram-negative bacterial strains (Liu et al., 2011; Chen et al., 2014; Romero et al., 2020). The antimicrobial activity of GO is mainly due to cell wrapping/trapping, insertion, damage of cell membrane, and generation of oxidative stress (Zou et al., 2017; Pulingam et al., 2019; Chen et al., 2021; Ravikumar et al., 2022). Our recent study demonstrated the correlation of time-dependent bacteriostatic and bactericidal effects of GO with changes in the cell membrane and expression of stress-associated proteins (Ravikumar et al., 2022). However, the antimicrobial intensity of pristine GO alone is not sufficient to kill bacterial cells on a large scale and inside bacterial communities such as biofilms (Pandit et al., 2021). This is limiting the application of GO in clinical settings, for the treatment of infectious lesions or prevention of bacterial biofilm formation on biomedical devices (Fallatah et al., 2019; Di Giulio et al., 2020). The antimicrobial performance of GO is heavily affected by the size, shape, thickness, surface charge, purity, and as well as dispersion status of GO nanosheets (Liu et al., 2012; Perreault et al., 2015; Barbolina et al., 2016; Anand et al., 2019). Some of these properties can be improved, and there exists considerable interest in combining GO with other nanomaterials or antimicrobial compounds to enhance its antimicrobial activity (Bugli et al., 2018; Singh et al., 2019; Han et al., 2020; Shamsi et al., 2022).

The cyclic cationic peptide polymyxin B (PMB), which is specific for Gram-negative bacteria, has been used as a “last resort” antimicrobial (Goode et al., 2021; Maynard et al., 2021). The antimicrobial activity of PMB is associated with cell membrane damage, inhibition of respiration, generation of reactive oxygen species, and inhibition of cell division (Mortensen et al., 2009; Trimble et al., 2016; Brennan-Krohn et al., 2018; Ayoub Moubareck, 2020). Specifically, in Gram-negative bacteria, the cationic peptide region of PMB binds electrostatically to the negatively charged lipopolysaccharide of the outer membrane (Fernández et al., 2013; Ayoub Moubareck, 2020; Manioglu et al., 2022). The hydrophobic fatty acid chains then interact with lipid A of the lipopolysaccharides, resulting in loss of membrane stability, facilitating the uptake of PMB into the outer membrane (Landman et al., 2008; Trimble et al., 2016). This phenomenon ultimately results in the weakening of the outer membrane and alters cell permeability. The disruption in cell membrane permeability results in the release of periplasmic proteins, damaging the cells (Yu et al., 2015; MacNair et al., 2018). In addition to affecting the membrane, it has been demonstrated that PMB exposure can generate reactive oxygen species (ROS) including superoxide, hydrogen peroxide, and hydroxyl radicals (Kohanski et al., 2007; Ayoub Moubareck, 2020). ROS causes oxidative damage of cellular components such as lipids, nucleic acids, and proteins, leading to cell death. We hypothesize that PMB combing with GO could be able to enhance the antimicrobial potential of graphene oxide. PMB was chosen due to its activity against Gram-negative strains that are resistant to other antibiotics, and due to its mode of action.

In this study, the natural PMB produced by *Bacillus polymyxa* (sulfate salt containing PMB B1 and B2. PMB B1 and PMB B2 contain an ethyl and a methyl group in the lipid chain, respectively) (Meng et al., 2016) was physisorbed onto the surface of pristine or functionalized GO (Guo et al., 2022). As GO colloidal stability decreased after PMB complexation, the surface of GO was modified by exploiting the epoxide ring opening to graft the amino triethylene glycol (TEG) chain. We describe the synthesis and characterization of GO and TEG-functionalized GO in combination with PMB, and we assess the capacity of the resulting materials to eradicate bacterial biofilms. Adsorbing PMB to the surface of GO drastically improved the bactericidal properties of GO, including its capacity for the destruction of biofilms made by Gram-negative pathogens *Pseudomonas aeruginosa* and *Escherichia coli*.

## 2 Materials and methods

### 2.1 Materials

GO was synthesized following a modified Hummers’ method (Morimoto et al., 2016) and obtained as an aqueous dispersion with a concentration of 3 mg/mL. The solvents were obtained from commercial suppliers and used without purification. Water was purified using a Millipore MilliQ^®^ filter system equipped with the free endotoxin Polisseur Biopak. When stated, the suspensions were sonicated in a water bath Elmasonic P sonicator with settings at 20 W and 40 kHz. MWCO 12,000-14,000 Da dialysis membranes were purchased from Spectrum Laboratories, Inc. Polymyxin B sulfate salt (mixture of polymyxin B1 and B2) and 2,2′-(ethylenedioxy)bis(ethylamine) were purchased from Sigma Aldrich. Depending on the types of containers employed, either 50 mL conical tubes or 1.5 mL microtubes, two centrifuges (Eppendorf Centrifuge 5804R or Eppendorf Centrifuge 5415R) were used.

### 2.2 Functionalization of graphene oxide and antibiotic adsorption

#### 2.2.1 Synthesis of GO-TEG

GO flakes were dispersed at a concentration of 1.43 mg/mL in endotoxin-free MilliQ^®^ water for the functionalization with 2,2′-(ethylenedioxy)bis(ethylamine) (TEG). The GO dispersion (7 mL) was stirred with Ultra-turrax t10 at low speed to reduce the agglomeration of the flakes during the functionalization process (Reina et al., 2019). TEG (3mg) in endotoxin-free MilliQ^®^ water (3 mL) was added dropwise using a pipette to reach a final concentration of GO of 1 mg/mL. After the TEG addition, the reaction was further magnetically stirred for 24 h. The unreacted TEG was eliminated by dialysis using endotoxin-free MilliQ^®^ water for 2 days. X-ray photoelectron spectroscopy (XPS), Thermogravimetric analysis (TGA), and Kaiser test analyses were used to determine the loading of TEG (Kaiser et al., 1970). Although this test is commonly employed in peptide synthesis, it has been adapted for use in carbon compounds (Ménard-Moyon et al., 2011; Jarre et al., 2014).

#### 2.2.2 Complexation with polymyxin B

Ultra-turrax t10 was used to stir GO and GO-TEG suspensions in Milli-Q^®^ at low speed (power 3) followed by the addition of a mixture of PMB B1 and B2 diluted in MilliQ^®^ water solution to reach a mass ratio of GO/PMB at 1:1. To allow the complexes to form, the suspensions were left under stirring at room temperature for 12 h. Then, the dispersions were centrifuged at 5000 rpm and washed with MilliQ^©^ water several times until the supernatant contained no PMB (as analyzed by HPLC). The clean conjugates (GO-TEG-PMB and GO-PMB) were freeze-dried. XPS and TGA analyses were used to determine the loading of PMB.

### 2.3 Characterization of materials

Morphological analyses were performed using a transmission electron microscope (Hitachi 7500, Hitachi High Technologies Corporation, Tokyo, Japan). The images were analyzed with FIJI software. TGA was performed on a TGA1 (Mettler Toledo) apparatus from 30°C to 900°C with a ramp of 10°C/min under N_2_ using a flow rate of 50 mL/min and platinum pans. For GO materials, samples were lyophilized before analysis. XPS analysis was performed on a Thermo Scientific K X-ray photoelectron spectrometer with a basic chamber pressure of 10^-8^-10^-9^ bar and an Al anode as the X-ray source (1486 eV). The samples were analyzed as powder pressed onto a scotch tape (3MTM EMI Copper Foil Shielding Tape 118). Spot size of 400 μm was used for analysis. The survey spectra were an average of 10 scans with a pass energy of 200 eV and a step size of 1 eV. For each sample, the analysis was repeated three times. A flood gun was turned on during the analysis. We grouped the functional groups of C1s spectra to avoid imprecision due to the proximity of the peak values and the overlapping of some bounds. Therefore, the C1s spectra were deconvoluted in C=O (287.6-289.9 eV) for carbonyl groups, C-O (286.2-287.2 eV) for hydroxyl and epoxide groups, C-N (285.9-286.2 eV) for the amine group and sulfur-containing groups and C-C (284.4-285.3 eV) for sp^2^ and sp^3^ carbon atoms. For the C-N peak, a range of error was considered since the binding energy of this bond overlaps with the C-O and C-C areas. For data analysis, CasaXPS (2.3.18) software was used. A Shirley background subtraction was applied. A line shape of 70% Gaussian/30% Lorentzian [GL(30)] was selected for all peaks. The FWHM was constrained to be the same for all peaks, apart from the π-π* peak because it is a broad signal. The N1s spectra were deconvoluted into two main peaks at 399.5 eV (N-R_2_) for the amine and 401.5 eV (N^+^-R_3_) for the ammonium. Infrared spectra of GO were recorded using an attenuated total reflectance Fourier transform infrared spectroscopy (ATR-FTIR) Alpha spectrometer from Bruker, with a diamond crystal as a refractive element, in the range 500-4000 cm^-1^ at a resolution of 4 cm^-1^.

### 2.4 Assessing the stability of GO-TEG-PMB complexation

Two mg of solid GO-TEG-PMB was dispersed into 1 mL of fresh LB medium (sonication bath; Elmasonic P30H,37 kHz). The solution was split into two microcentrifuge tubes (1.5 mL) and kept in the dark at 37°C for 1 day for one of the tubes and 7 days for the other tube. Each microtube was centrifuged at 12000 rpm for 15 min and the supernatant was analyzed by HPLC. HPLC analyses were performed on a Waters Alliance e2695 separations module instrument equipped with an autosampler and 2998 PDA detector, on a Nucleosil C18 column (150×4.6 mm), using a 20 min linear gradient of 0.1% TFA in water to 31% or 100% of 0.08% TFA in acetonitrile at a flow rate of 1.2 mL·min^-1^.

### 2.5 Antimicrobial activity of the functionalized GO-PMB complex

Minimum inhibitory concentration (MIC) and minimum bactericidal concentration (MBC) of tested agents were determined by microdilution method as described previously (Cai et al., 2015). Briefly, overnight grown cultures of *P. aeruginosa* and *E. coli* were appropriately diluted, and 100 μL of diluted bacterial suspensions were mixed with a serial dilution of nanomaterials. The bacterial concentration in the mixtures was 1–2×10^5^ colony-forming units (CFU)/mL, and the concentration of tested agents ranged from 1.56 to 100 μg/mL in a series of twofold dilutions. The samples were incubated at 37°C for 24 h, and optical density at 600 nm (OD_600_) was measured. The MIC was defined as the lowest concentration of tested agent which inhibited bacterial growth. For defining the MBC, 10 μL of mixtures described earlier (bacteria and nanomaterials) were placed on agar plates and incubated at 37°C overnight. MBC values were defined as the lowest concentration of the tested agent which prevented the growth of bacteria on agar plates. The treatment time dependent bactericidal efficiency of GO, GO-TEG, and PMB complexed to the different GO was tested against the planktonic cells of *P. aeruginosa* and *E. coli*. To prepare a bacterial inoculum, single colonies of *P. aeruginosa* and *E. coli* were inoculated to 5 mL of LB medium and incubated overnight at 37°C with continuous agitation. For this assay, 20 μL of inoculum (2–5×10^7^ CFU/mL bacterial cells) from overnight grown bacterial cultures were inoculated into fresh LB medium containing sterile deionized water (control), MIC, and 2 times MIC concentration of tested agents. All samples were incubated at 37°C with continuous agitation for 24 h. A fraction of culture (100 μL) from each sample was taken to determine bacterial viability at time points of 0, 4, 8, and 24 h. The collected samples were serially diluted in 0.89% of a saline buffer. From the diluted samples, 100 μL were plated onto LB agar plates, incubated at 37°C for 24 h, and the number of colonies was counted.

### 2.6 Antibiofilm activity of GO-PMB complexes

Biofilms of *E. coli* and *P. aeruginosa* were formed on 15 mm cover glass. Briefly, overnight grown bacterial culture was diluted with fresh LB to make final inoculum 2×10^6^ –5×10^6^ CFU/mL. 300 μL of bacterial inoculum were loaded to cover glass and incubated at 37°C without any disruption to achieve biofilm formation. After 24 h, the culture medium was replaced with fresh LB medium (control) or MIC and 2×MIC of tested agents, and the samples were further incubated for 24 h. After 24 h of treatment, biofilms were collected in 5 mL of 0.89% of NaCl and homogenized by sonication. The homogenized biofilm suspensions (100 μL) were serially diluted and plated on LB agar plates and incubated overnight at 37°C. The number of colonies was counted to determine the viability of bacterial cells (CFUs). To visualize the live and dead cells, control biofilms and biofilms treated with test agents were stained for 20 min with a mixture of 6.0 μM SYTO 9 and 30 μM propidium iodide from a live/dead BacLight Viability kit L13152 (Thermo Fisher Scientific). Fluorescence microscopic imaging of the biofilms was performed using a Zeiss fluorescence microscope (Axio Imager.Z2m; Carl Zeiss Meditec AG, Jena, Germany). Scanning electron microscopy (SEM) analysis of biofilms was performed as described previously (Pandit et al., 2020). Briefly, biofilms were fixed with 3% of glutaraldehyde for 2 h and dehydrated with graded series of ethanol concentrations (40%, 50%, 60%, 70%, 80%, and 90% for 15 min each) and with absolute ethanol for 20 min. The dehydrated biofilm samples were dried at room temperature overnight and coated with gold before SEM imaging, performed with a Supra 60 VP microscope (Carl Zeiss AG, Jena, Germany).

### 2.7 Testing GO- PMB complexes as catheter coating

In order to deploy functionalized GO-PMB as a surface coating, a commercially available catheter was purchased, sliced into pieces (12 mm) and sonicated for 2 h with the sterile water containing 100 µg/mL of GO and GO-TEG, 2×MIC concentration of PMB complexed to GO and GO-TEG (Sonication bath, 100% of amplitude). The coated catheter tubes were dried, and UV sterilized before testing the biofilm formation. The inoculum for biofilm formation was prepared by using overnight grown culture of *P. aeruginosa* and *E. coli* and it was diluted with fresh LB to make the final inoculum 2×10^6^ –5×10^6^ CFU/mL. The coated and non-coated catheter tubes were individually placed in culture tube containing 2 mL of inoculum. The culture tubes were incubated at 37°C for 24 h. After 24 h, biofilm on catheter tube was dip washed using sterile water and fixed with 3% of glutaraldehyde for 2 h. After fixation samples were dehydrated with graded series of ethanol concentrations (40%, 50%, 60%, 70%, 80%, and 90% for 15 min each) and with absolute ethanol for 20 min. The dried samples were sputter coated with gold and analyzed by SEM imaging.

### 2.8 Statistical analysis

All microbiology data are presented as the mean ± standard deviation from at least three different biological replicates. Intergroup differences were estimated by one-way analysis of variance (ANOVA), followed by a *post hoc* multiple comparison (Tukey) test to compare the multiple means. Differences between values were considered statistically significant when the *P*-value was < 0.05.

## 3 Results

### 3.1 Synthesis and characterization of functionalized GO

**Figure 1:**
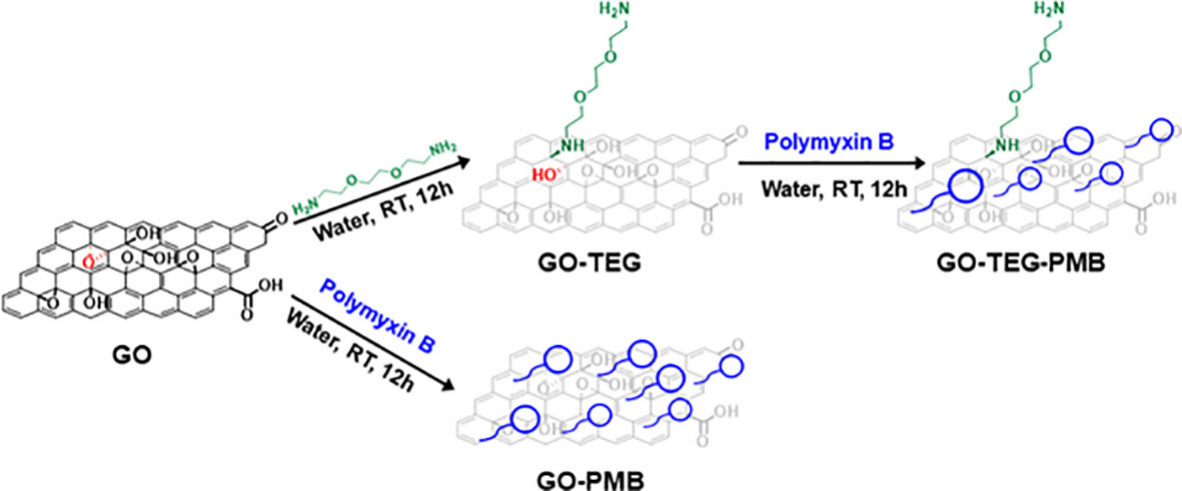
Schematic illustration of the processes to prepare GO-based materials combined with PMB. PMB is complexed to the two GO-based materials either after functionalization of GO with the TEG linker or directly to GO.

The starting GO was obtained from a modified protocol of Hummers oxidation of graphite, leading to a stable aqueous dispersion at the concentration of 3 mg/mL. According to TEM images, single layer GO had an average size of 1.49 ± 0.9 µm (**Figure S1**). The adsorption of PMB on the surface of GO was carried out at room temperature by mixing the two components in aqueous solutions (**Figure 1**). To achieve a homogenous adsorption, the mixture was exposed to an ultraturrax-generated vortex during the addition of the peptide to GO. The HPLC analysis of collected supernatant from washing process demonstrates the absence of free PMB (**Figure S2**). The complex was subsequently freeze-dried to maintain its structure and prevent peptide release in the solution during storage. After redispersion of the complex in an aqueous solution, its colloidal stability was reduced compared to GO alone (**Figure S3**).

To improve the stability of the dispersion, GO was modified by covalent functionalization with a short polyethylene glycol (PEG) chain to promote the dispersion of materials and particles in an aqueous environment. The primary amines of NH_2_(CH_2_CH_2_O)_2_CH_2_CH_2_NH_2_ (TEG) were allowed to react with the epoxide rings present onto the surface of GO (**Figure 1**). This approach of GO functionalization is an easy and practical method to reach a high level of functional groups under mild conditions. Verification and quantification of the amino functions were performed after the purification of GO-TEG by dialysis using the Kaiser test. The concentration of the amines resulted in 442 µmol/g of GO. The order of magnitude is similar to that calculated by XPS (577 µmol/g of GO) (*vide infra*). Then, PMB was adsorbed onto the surface of GO-TEG under the same condition used for non-functionalized GO. TEG functionalization considerably increased the complex dispersion in an aqueous solution (**Figure S3**). In addition to HPLC, the different complexes were also characterized by TGA, XPS, and FTIR.

**Figure 2:**
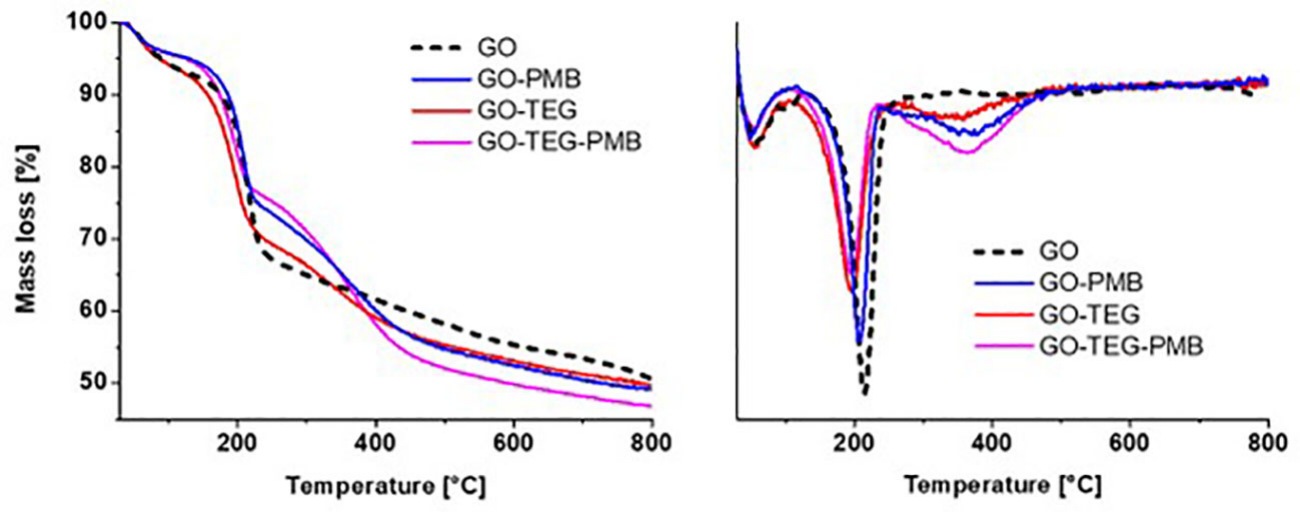
Left) Thermogravimetric analysis of GO, GO-PMB, GO-TEG, and GO-TEG-PMB. Right) Derivative thermogravimetry of GO, GO-PMB, GO-TEG and GO-TEG-PMB. The differences in the curves of the weight loss and the shifts in the position of derivative minima indicate the occurrence of the functionalization of GO and the complexation with PMB.

TGA, conducted under an inert atmosphere, allowed us to evaluate the thermal degradation profile of GO conjugates (**Figure 2**, left). In the temperature range between 30 and 800°C, GO conjugates lost more weight than pristine GO. Calculation on the derivative thermogram (**Figure 2**, right) revealed that GO exhibits the typical two-step degradation profile, with a first modest step at around 100°C and a second one at around 200°C, associated with the loss of water and labile oxygenated groups, respectively. After the loss of water and labile oxygenated groups, an additional step could be observed between 300-400°C, depending on the type of functionalities added to the material surface. This additional step in the thermal profile can be attributed to polymer degradation for GO-TEG and polypeptide PMB degradation for GO-PMB and GO-TEG-PMB, respectively. In addition, there is also a noticeable shift in the step of degradation around 200°C for materials containing TEG chains (**Figure 2**, right). This shift is caused by a change in the surface chemistry of GO caused by the covalent functionalization, which opened the epoxide ring and introduced secondary amines (less thermally stable). The different functionalization steps increased the weight of the compounds, which translates into a greater loss of mass observable above 450°C for the different conjugates, following this trend: GO-TEG (+2.2% compared to GO), GO-PMB (+2.8% compared to GO) and GO-TEG-PMB (+3.3% compared to GO-TEG).

**Figure 3:**
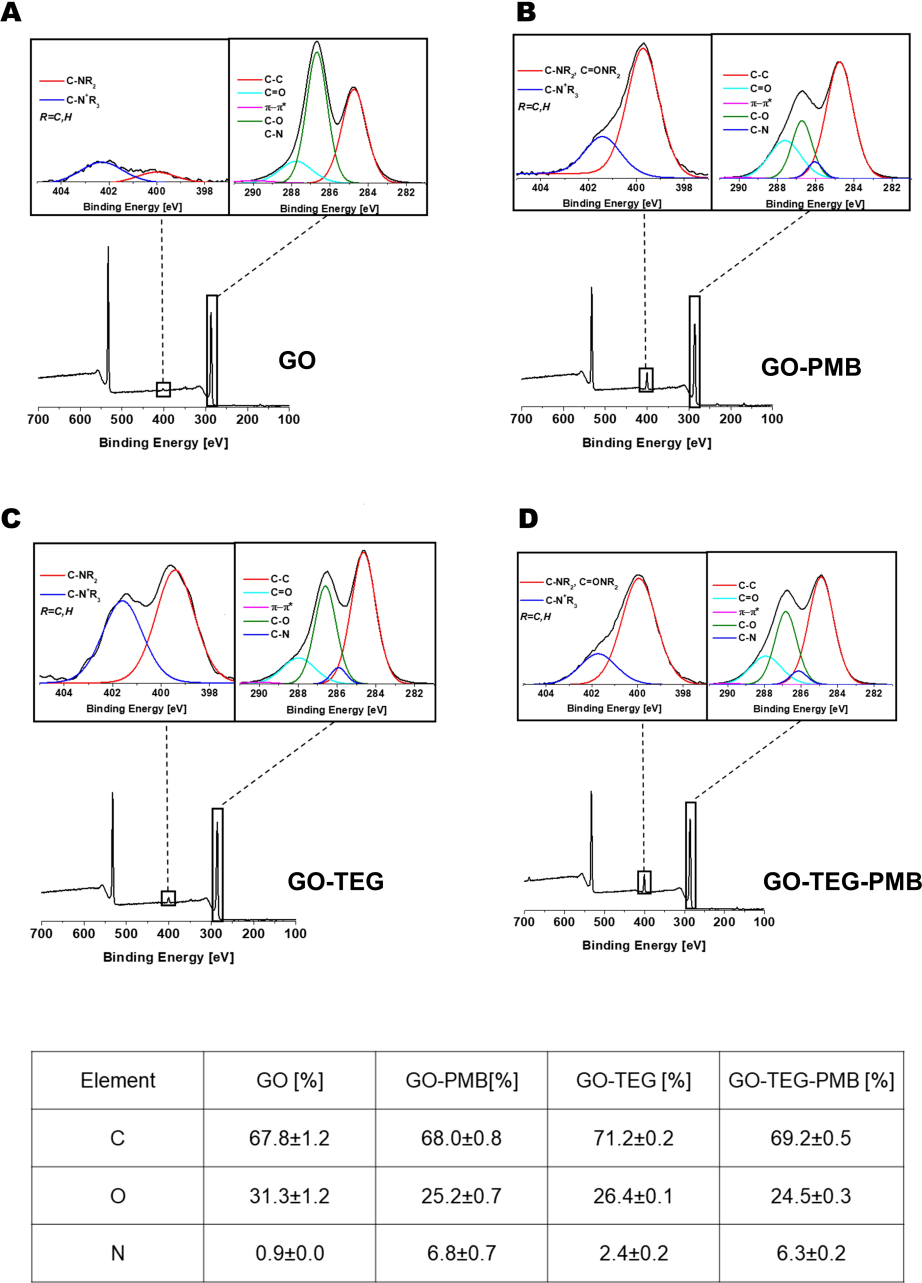
XPS characterization. XPS survey spectra with high resolution N1s (left inset) and C1s (right inset) of GO (panel **(A)**, GO-PMB (panel **(B)**, GO-TEG (Panel **(C)** and GO-TEG-PMB (Panel **(D)**. Bottom: table reporting the relative XPS atomic percentage of C, O, and N.

**Figure 4:**
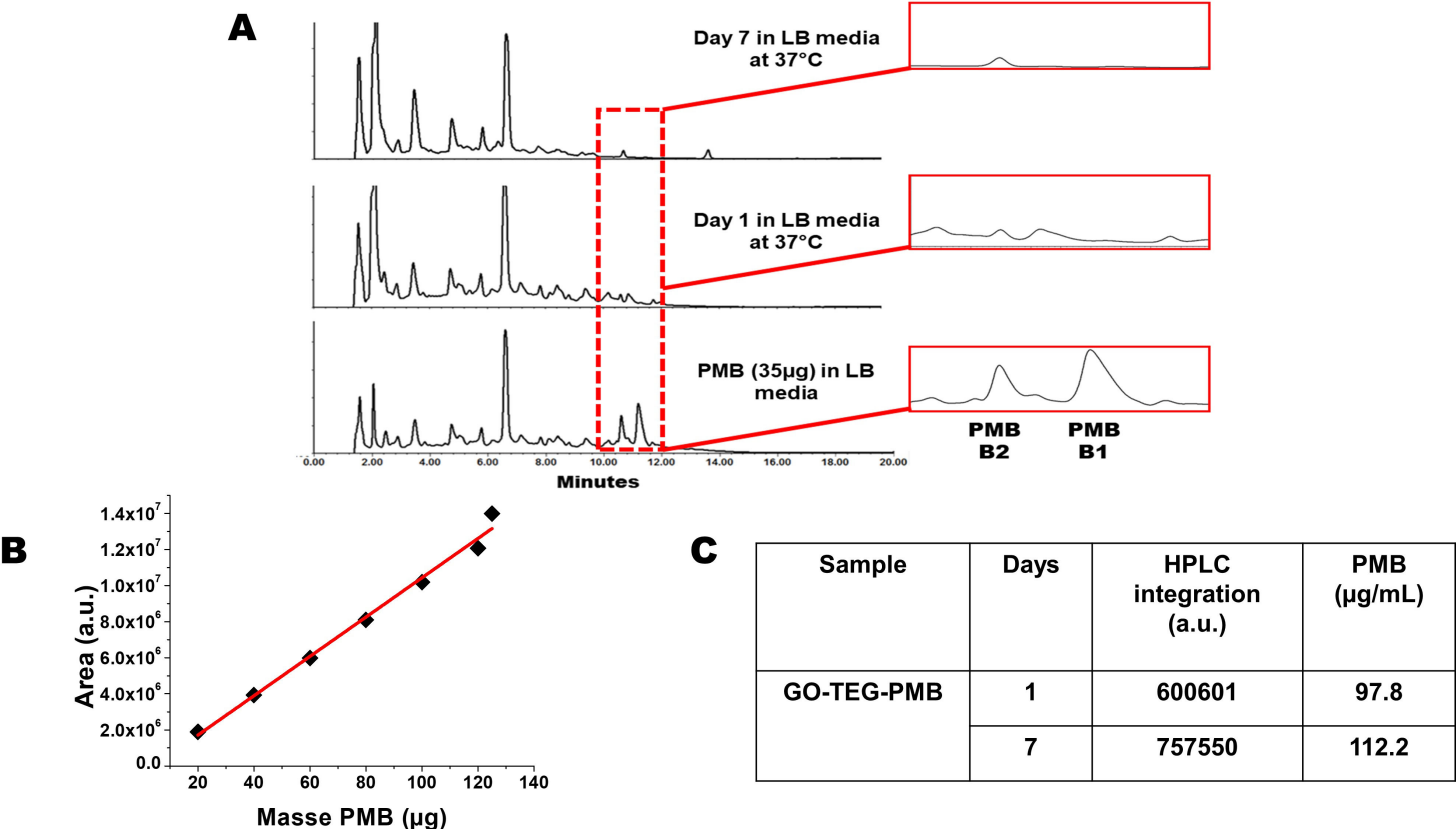
**(A)** HPLC chromatograms of PMB and GO-TEG-PMB stability in LB culture media incubated for 1 day and 7 days at 37°C. **(B)** Calibration curve using different amounts of PMB injected in HPLC. **(C)** Table reporting the different integrated areas of PMB peaks released from GO-TEG-PMB on day 1 and day 7.

Subsequently, XPS was performed to study the surface composition and chemical state of the different GO conjugates before and after the complexation with PMB (**Figure 3**). The addition of the TEG or PMB resulted in clear changes in the XPS spectra, as expected. In comparison to the pristine GO, which was mostly composed of C and O atoms, the addition of TEG and/or PMB increased the number of N atoms. The N1s spectra of functionalized GO show two peaks, at 399.6 and 401.6 eV, corresponding to amines and positively charged amines, respectively. In the case of the high-resolution C1s peaks of the different conjugates, the contribution of C-O bonds is reduced in comparison to pristine GO, implying that the introduction of TEG chain or the peptide increases the ratio of C-C bonds. Furthermore, in comparison to the initial GO, a novel contribution to the C1s deconvolution peak at 286.0 eV was identified. Even if this contribution overlaps with the value of the C-OH binding energy, the appearance of this one is caused by the formation of the bond C-N on the surface during the opening of the epoxides by the primary amine of the chain TEG, and by the presence of the PMB, which contains many amide bonds and the unnatural amino acid 2,4-diaminobutyric acid. The analysis of the XPS surveys allowed us to calculate the number of functional groups. The initial content of nitrogen on GO was 0.9%, and the addition of PMB and TEG considerably augmented this amount (Table at the bottom of **Figure 4**). Considering the molecular weight of each function, the number of N atoms in the molecules, and the value obtained from the starting GO, the loading of TEG was calculated to be 577 µmol/g of GO (8.5 wt%) for GO-TEG. The loading of PMB corresponded to 281 µmol/g of GO (39 wt%) for GO-PMB, and 228 µmol/g of GO (32 wt%) for GO-TEG-PMB.

Finally, we analyzed the FT-IR spectra of the different GO conjugates to confirm the covalent functionalization with TEG as well as the coupling with PMB (**Figure S4**). In the spectrum of the starting GO, the broad peak around 3400 cm^-1^ was assigned to the O-H stretching of the adsorbed water and the hydroxyl functions of GO. The band at 1723 cm^-1^ in GO was attributed to the stretching of C=O groups, while the peak at 1619 cm^-1^ corresponded to the H-O-H bending vibration of water molecules and the skeletal C=C bond vibrations of the graphitic domains. The band at 1371 cm^-1^ could be assigned to the O-H bending vibration. The C-O-C vibration band of the epoxides was located at 1232 cm^-1^ and the peak at 1143 cm^-1^ was assigned to the C-O stretching. The presence of amide I and amide II bands in conjugated GO-PMB and GO-TEG-PMB supports the existence of adsorbed PMB onto the surface of GO. The two peaks located at 1647 cm^-1^ and 1533 cm^-1^ in free PMB can also be found in GO-PMB and GO-TEG-PMB.

### 3.2 Release of PMB from functionalized GO

Our choice of non-covalent complexation between GO and PMB can raise the issue of uncontrolled release of the peptide during the dispersion in the cell culture media and incubation with bacteria. In this context, we investigated the stability of our GO-TEG-PMB conjugate over time. The material was dispersed in LB culture media using a sonication bath, to prepare two dispersions of 500 µL at 2 mg/mL of GO-TEG-PMB. The dispersions were placed in the incubator at 37°C. The first mixture was centrifuged after 1 day and the second one after 7 days. The supernatants were collected and analyzed by HPLC (**Figure 4A**). A calibration curve with PMB at different concentrations in LB medium was performed (**Figure 4B**) to calculate the quantity of PMB released over time (**Figure 4C**). The chromatogram peaks between 10 and 12 min correspond to PMB identified in LB medium. The first belongs to PMB B2 and the second to PMB B1. The two compositions of PMB differ by the terminal fatty acid chain, with PMB B2 possessing a methyl group, while in PMB B1 the analogue chain is an ethyl group. The presence of PMB B2 in the medium is also visible in the chromatograms of the two supernatants at day 1 and day 7. The analogue with the longer fatty acid chain (B1) seems to remain adsorbed more strongly onto the surface of GO than PMB B2, which is mainly released after 24 h. The calculations allowed to establish a release of 98 µg per 1 mg of GO-TEG-PMB after 24 h and 112 µg after 7 days, corresponding to 10% and 11% of release, respectively. On the basis of these data, we can conclude that almost all PMB B2 is rapidly detached from the surface of GO by comparing the ratios of PMB B1 and PMB B2 (3:1), as well as the previously estimated loading (0.32 g of PMB for 1 g of conjugate).

### 3.3 PMB adsorption significantly enhances the bacteriostatic and bactericidal efficiency of GO

The antimicrobial efficacy of GO and PMB-incorporated GO was first characterized by MIC and MBC values against planktonic *E. coli* and *P. aeruginosa*. As shown in **Table 1**, the MIC and MBC of pristine GO and TEG functionalized GO were > 100 µg/mL, hence we did not document any antimicrobial effect of GO alone (**Table 1**). Bacteriostatic and bactericidal efficiency was established after PMB adsorption. The bacteriostatic effect of GO-PMB was stronger than GO-TEG-PMB against *P. aeruginosa*. There was no difference in the bactericidal efficiency of GO-PMB and GO-TEG-PMB against both tested bacterial strains (**Table 1**).

**Table 1:** MIC and MBC of GO, GO-TEG, GO-PMB and GO-TEG-PMB against P. aeruginosa and E. coli.

P. aeruginosa			E. coli		
Material	MIC (µg/mL)	MBC (µg/mL)	Material	MIC (µg/mL)	MBC (µg/mL)
GO	> 100	> 100	GO	> 100	> 100
GO-TEG	> 100	> 100	GO-TEG	> 100	> 100
GO-PMB	25	50	GO-PMB	50	50
GO-TEG-PMB	50	50	GO-TEG-PMB	50	50

**Figure 5:**
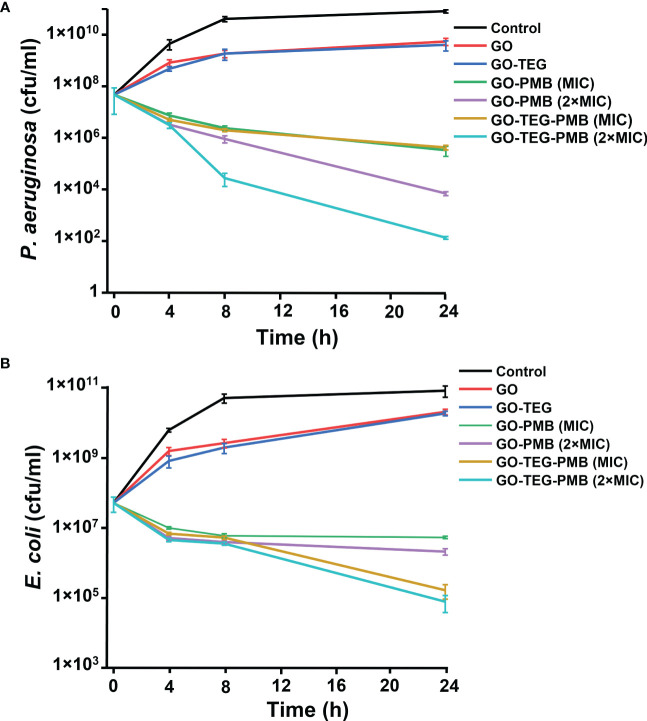
Time kills assay of tested agents against the planktonic culture of *E. coli* and P. aeruginosa. CFU counts of **(A)** P. aeruginosa and **(A)** *E. coli* exposed to GO (100 μg/mL), GO-TEG (100 μg/mL), GO-PMB, and GO-TEG-PMB with respect to treatment time. Both bacterial strains are grown in standard cultivating conditions. Data are presented as mean ± standard deviation from three independent biological replicates.

Based on MIC and MBC values, concentrations of test agents were selected for time kill assays gain more detailed insight into the antimicrobial effect. GO and GO-TEG did not fully inhibit the growth of *E. coli* and *P. aeruginosa*, but the cell concentration was 10 folder lower than the untreated controls after 24 h incubation (**Figure 5**). By contrast, the treatment with GO-TEG-PMB significantly reduced the number of viable bacterial cells already after 4 h of incubation. The concentration-dependent bactericidal activity of GO-TEG-PMB was also clearly visible in the time killing assay after 24 h of treatment. Consistent with the MIC and MBC results, *P. aeruginosa* was more sensitive to the tested agents than *E. coli*. The viability of *P. aeruginosa* was drastically decreased by GO-PMB (3 log CFU) and GO-TEG-PMB (5 log CFU) after 24 h of treatment at 2×MIC concentration of tested agents (**Figure 5A**). In comparable conditions, the viability of *E. coli* was reduced only by 1 and 2 log of CFUs, respectively, by GO-PMB and GO-TEG-PMB (**Figure 5B**).

### 3.4 GO-TEG-PMB treatment destroys preformed bacterial biofilms

**Figure 6:**
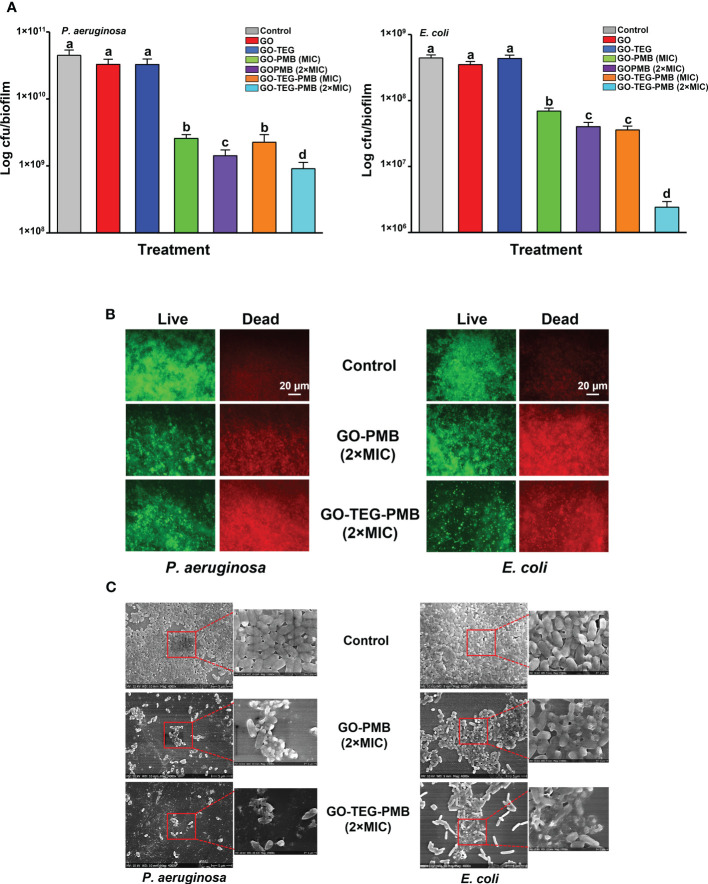
Measurement of antibiofilm activity of GO, GO-TEG, GO-PMB, and GO-TEG-PMB against **(A)** *P. aeruginosa* and *E. coli*. 24 h old biofilms were exposed to fresh medium containing test agents for an additional 24 h, and the viability of bacteria was expressed as CFUs. Data represents mean ± standard deviation from three independent biological replicates. Values labeled by the different superscript are significantly different from each other (P < 0.05). **(B)** Live/dead viability staining of biofilms of *P. aeruginosa* and *E. coli* after 24 h of treatment. Representative fluorescence microscopy images from three independent biological replicates are presented. Green color denotes live bacteria and red color denotes dead bacteria. **(C)** Representative SEM images of *P. aeruginosa* and *E. coli* biofilms after 24 h of treatment.

**Figure 7:**
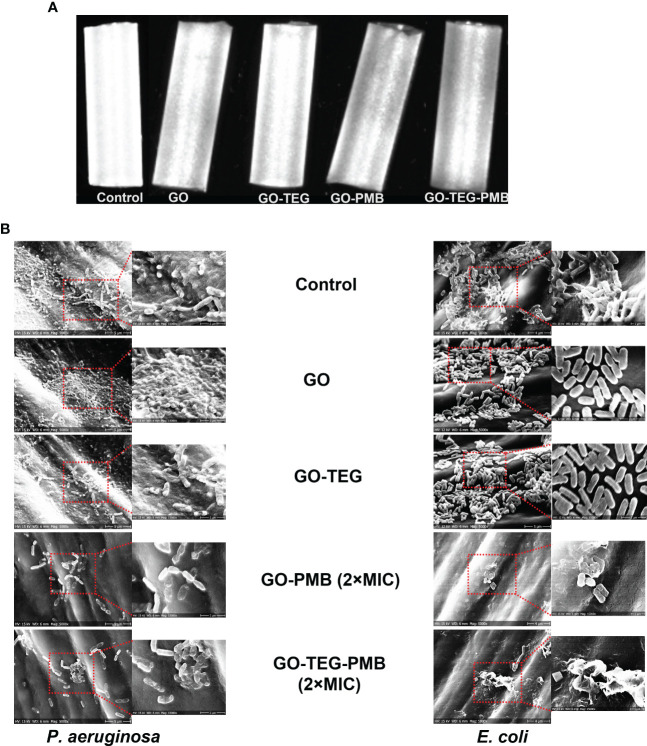
**(A)** Photograph of GO, GO-TEG, GO-PMB and GO-TEG-PMB coated polymer-based catheter tubes. **(B)** Representative scanning electron microscopy images of P. aeruginosa and *E. coli* biofilm formation on coated and non-coated catheter tubes after 24 h of bacterial cultivation.

In order to determine the antibiofilm activity, preformed 24 h old biofilms of *E. coli* and *P. aeruginosa* were treated with all the tested materials. After the 24 h of treatment with each tested agent, antibiofilm activity was examined by means of CFU counting, live/dead viability staining, and scanning electron microscopy (**Figure 7**). The viability of both *E. coli* and *P. aeruginosa* biofilms were not significantly affected by GO and GO-TEG. By contrast, GO-PMB and GO-TEG-PMB significantly altered the viability of biofilms of both tested stains. Furthermore, the reduction of a number of biofilm cells was observed to be concentration dependent, since 2×MIC reduced the viability of bacteria significantly higher than 1×MIC (**Figure 6A**). The antibiofilm effect of GO-TEG-PMB was significantly stronger than that of GO-PMB. Interestingly, the increase of the GO-TEG-PMB concentration to 2×MIC was more effective against the *E. coli* biofilms. This does not follow the trend observed with planktonic bacterial cultures. This difference is likely due to the difference in density and composition of the biofilms matrix of respective bacterial strains, reflecting on their resilience. To confirm the results obtained from CFU counting, control, and treated biofilms were stained with live/dead viability staining and examined using a fluorescence microscope. **Figure 6B** shows the representative images acquired after the live/dead staining where green color depicts the live cells and red color depicts the dead cells. A higher density of dead cells was observed in biofilms treated with 2×MIC of GO-PMB and GO-TEG-PMB compared to controls. Both *P. aeruginosa* and *E. coli* biofilms were more strongly affected by GO-TEG-PMB (green to red ratios should be compared). These observations correlated well with the findings from CFU counting. Furthermore, to examine the effect of the treatment on the architecture of the biofilm matrix and cell morphology, biofilms were examined by SEM (**Figure 6C**). Both GO-PMB and GO-TEG-PMB were found to significantly alter the biofilm organization and deactivate bacterial cells by disrupting the cell membranes. Both tested materials at a 2×MIC clearly destroyed the biofilms of both *P. aeruginosa* and *E. coli*, leaving either a fraction of altered microcolonies or single cells disconnected from the complex community. Most of the visible cells in such microcolonies or individual cells were either morphologically altered or collapsed due to cell membrane damage leading to leakage of intracellular components.

### 3.5 GO-TEG-PMB coating strongly mitigates biofilm formation on commercial catheters

Next, we asked whether the strong biofilm disruptive activity of GO-TEG-PMB can be useful in terms of preventing biofilm formation on typical biomedical devices. To test that, small pieces of catheter tubes were coated with individual test materials. Representative photographs of test agent coated catheters are presented in **Figure 7A**. The coated catheters were exposed to bacterial cultures (24 h) for biofilm formation and the adhesion of bacterial cells on the catheter surfaces was examined by SEM (**Figure 7B**). As shown in SEM images, bacterial adhesion and biofilm formation of both *P. aeruginosa* and *E. coli* on the surface of pristine GO- and GO-TEG-coated catheters appear to be similar to the control non-coated surface. By contrast, bacterial adhesion was drastically reduced on catheters coated with GO-PMB and GO-TEG-PMB. In addition to preventing bacterial adhesion, GO-PMB and GO-TEG-PMB coatings seemed to also disrupt the adhered bacterial cells.

## 4 Discussion

The goal of this study was to improve the efficacy of GO as an antibacterial agent against Gram-negative biofilms. To achieve this, several aspects were taken into consideration. One of the strategies is based on a combined antibacterial effect of GO and PMB. Previous studies have reported that the lateral size of GO should be around one micrometer (Liu et al., 2011; Liu et al., 2012) for optimal intrinsic effectiveness of the material against bacteria. GO sheets possessing this lateral size can fully cover the bacterial membrane by wrapping the cells, thus inhibiting bacterial metabolic activity and isolating them from the surrounding environment (Akhavan et al., 2011). However, size is not the only factor to consider when designing antibacterial GO sheets. Another parameter to consider is the level of oxidation. According to the strategy we adopted for our study, it was preferable to choose a highly oxidized GO, to allow for better molecular adsorption (Thangavel and Venugopal, 2014) and to boost the oxygenated functionalities for covalent modification of the surface. PMB was chosen because it is an effective antimicrobial agent used against multidrug-resistant bacteria (Zavascki et al., 2007; Siddiqui et al., 2014). We decided to complex pristine and functionalized GO with the antimicrobial peptide PMB. Since GO contains graphitic sp^2^ domains and areas rich in oxygenated functions (e.g., hydroxyl and epoxy groups), physisorption between GO and PMB can occur *via* π-π stacking, electrostatic forces, and hydrogen bonding.

In this study, PMB was mixed with GO and TEG functionalized GO in order to achieve adsorption onto surface of pristine and functionalized GO. The rationale of TEG functionalization was to add a hydrophilic chain to enhance the dispersibility of GO. The adsorption of PMB was confirmed by XPS and FTIR (**Figure 3**, **Figure S4**) and HPLC results confirmed gradual release of PMB from the complex material, with about 10% release after one day, and further 10% release up to day 7 (**Figure 4**). This indicated that coatings based on GO-TEG-PMB could provide sustained delivery of PMB and offer long-term antibacterial protection. From the physicochemical analyses of the complexes we can draw the following conclusions. The improvement in the dispersibility of the complex resulting from the mixture of GO and PMB was achieved through the insertion of a TEG chain *via* the formation of covalent bonds. This covalent linking occurs *via* the ring opening of the epoxides onto the surface of GO by the primary amine of TEG as demonstrated in previous articles (Vacchi et al., 2016). The interactions between the two GO materials and PMB are mainly electrostatic and hydrophobic, the latter likely due to the presence of the lipid chain on PMB, while π-π interactions are unlikely as there is only one phenylalanine in the peptide sequence. Indeed, we did not observe an increase of the π-π signals in the high resolution XPS C1s spectra.

The antimicrobial activity of the engineered materials was then evaluated by MIC and MBC assays. As expected, PMB adsorption brought down the MIC and MBC concentration of GO to 25 and 50 µg/mL (**Table 1**), respectively, suggesting the increase in antimicrobial potential. Time dependent killing assays further confirmed the bacteriostatic and bactericidal efficiency of pristine and PMB adsorbed GO. The strong time dependent bactericidal activity of GO-TEG-PMB can be due to the higher release of PMB compared to GO-PMB (**Figures 4C**, **5**). Despite exhibiting a similar bactericidal trend against both *P. aeruginosa* and *E. coli*, the killing efficiency of GO-TEG-PMB was higher against *P. aeruginosa*. This difference is most likely due to the strain difference in sensitivity to PMB and GO.

Most of the previous studies regarding the antimicrobial activity of pristine and functionalized GO were conducted with bacterial cells in a planktonic state (de Moraes et al., 2015; Karahan et al., 2016; Ye et al., 2017). However, in clinical as well as environmental settings, bacterial cells tend to adhere to surfaces and form 3-dimensional structure called biofilms (Kordmahaleh & Shalke, 2013). The bacterial communities in such biofilm matrix tolerate up to 1000 times higher concentrations of antimicrobial agents (Sharma et al., 2019). It is important to consider testing the potential of drug/nanomaterials against the biofilm cells if the agents are aimed at the treatment of infectious diseases. Thus, to examine antibiofilm potential, preformed biofilms were treated with GO, GO-PMB, and GO-TEG- PMB. The obtained results clearly demonstrated a concentration-dependent bactericidal efficiency of the tested materials against biofilms. Since pristine GO and GO-TEG showed no bactericidal activity, we would propose that the observed antibiofilm activity of GO-PMB and GO-TEG-PMB is mainly due to the release of PMB in contact with the biofilms. This difference in antibiofilm activity against different bacterial strains may be due to the difference in the composition and density of exopolymer matrix in the biofilms of *E. coli* and *P. aeruginosa*. Furthermore, the results from anti-biofilm activity of PMB adsorbed GO were confirmed by live/dead viability staining. The staining kit contains two nucleic acid stains SYTO 9 and propidium iodide. The fluorescence stain SYTO 9 can permeate the cell membrane, thus entering living cells and staining them in green. Propidium iodide cannot permeate the cell membrane, hence only the cells that are damaged and morphologically altered and stained in red. As presented in **Figure 6B**, there is an increase in the density of dead cells in the biofilms treated with GO-PMB and GO-TEG-PMB. While the live/dead fluorescence microscopy images can visualize the general trend of bactericidal efficiency of tested agents against biofilms, it cannot clearly visualize the intensity of damage to cells inside the biofilm matrix. To visualize this, treated biofilms were examined with SEM (**Figure 6C**). As suggested by the number of propidium iodide-stained cells, the treatment led to extensive damage or full collapse of the cell membranes in most of the visualized cells.

Prevention of biofilm formation is considered as an excellent approach for inhibition of biomedical device associated infections. Indeed, the adhesion of pathogenic microorganism to biomedical devices and subsequent biofilm formation is a great challenge and may cause severe infections (Lebeaux et al., 2014). Antimicrobial coatings using nanomaterials and composites are considered as one of the promising approaches to prevent biofilm formation on the surface of such devices (Pandit et al., 2018; Chen et al., 2020). To test the efficiency of PMB adsorbed GO in preventing biofilm formation, catheter tubes were coated with GO-TEG-PMB. This has led to a very clear reduction of bacterial adhesion and visible damage to the attached cells. These results suggested that coatings based on GO-TEG-PMB are truly applicable in clinical settings, with a clear potential for preventing biofilm formation on medical devices. In summary, we propose that our method for combing GO and PMB can lead to a material that is easy to produce, easy to apply as a coating on biomedical devices and offers strong and lasting protection against Gram-negative bacterial biofilms based on the sustained release of the antibacterial agent.

## Data availability statement

The original contributions presented in the study are included in the article/**Supplementary Material**. Further inquiries can be directed to the corresponding authors.

## Author contributions

Conceptualization, SP, IM, AB, and CP. Methodology, SP, LJ, JZ, ZG, YN, and CP. Validation, SP, LJ, JZ, ZG, YN, and CP. Data curation, SP, LJ, JZ, ZG, YN, and CP. Writing—original draft preparation, SP, LJ, and CP. Writing—review and editing, SP, RM, IM, AB, and CP. Project administration, SP, IM, AB, and CP. Funding acquisition, SP, CP, IM, and AB. All authors contributed to the article and approved the submitted version.
