# Supplementary material for: Polymyxin B complexation enhances the antimicrobial potential of graphene oxide
Source: Front Cell Infect Microbiol. 2023 Jun 21;13:1209563. doi: 10.3389/fcimb.2023.1209563 (PMC10321305; doi:10.3389/fcimb.2023.1209563)
Supplement: Supplementary file 1 [file DataSheet_1.docx]

**Polymyxin B complexation enhances the antimicrobial potential of graphene oxide**

**Santosh Pandit^1#^, Lucas Jacquemin^2#^, Jian Zhang^1^, Zhengfeng Gao^2^, Yuta Nishina^3,4^, Rikke Louise Meyer^5,6^, Ivan Mijakovic^1,7^, Alberto Bianco^2^*, Chengfang Pang^8,9^***

^1^ Systems and Synthetic Biology Division, Department of Life Sciences, Chalmers University of Technology, SE-412 96 Gothenburg, Sweden

^2^ CNRS, Immunology, Immunopathology and Therapeutic Chemistry, UPR 3572, University of Strasbourg, ISIS, 67000 Strasbourg, France

^3^ Research Core for Interdisciplinary Sciences, Okayama University, 3-1-1 Tsushimanaka, Kita-ku, Okayama 700-8530, Japan

^4^ Graduate school of natural science and technology, Okayama University, 3-1-1 Tsushimanaka, Kita-ku, Okayama 700-8530, Japan

^5^Interdisciplinary Nanoscience Center, Aarhus University, 8000 Aarhus C, Denmark

^6^ Department of Biology, Aarhus University, 8000 Aarhus C, Denmark

^7^ The Novo Nordisk Foundation, Center for Biosustainability, Technical University of Denmark, DK-2800 Kongens Lyngby, Denmark

^8^ Research Group for Genomic Epidemiology, National Food Institute, Technical University of Denmark, DK-2800 Kongens Lyngby, Denmark

^9^ The Intelligent Drug Delivery and Sensing Using Microcontainers and Nanomechanics, Department of Health Technology, Technical University of Denmark, DK-2800 Kongens Lyngby, Denmark

# Authors contributed equally to this work.

*** Correspondence:**Chengfang Pang

[chet@food.dtu.dk](mailto:chet@food.dtu.dk)

Alberto Bianco
[a.bianco@ibmc-cnrs.unistra.fr](mailto:a.bianco@ibmc-cnrs.unistra.fr)

# Supplementary Material

**
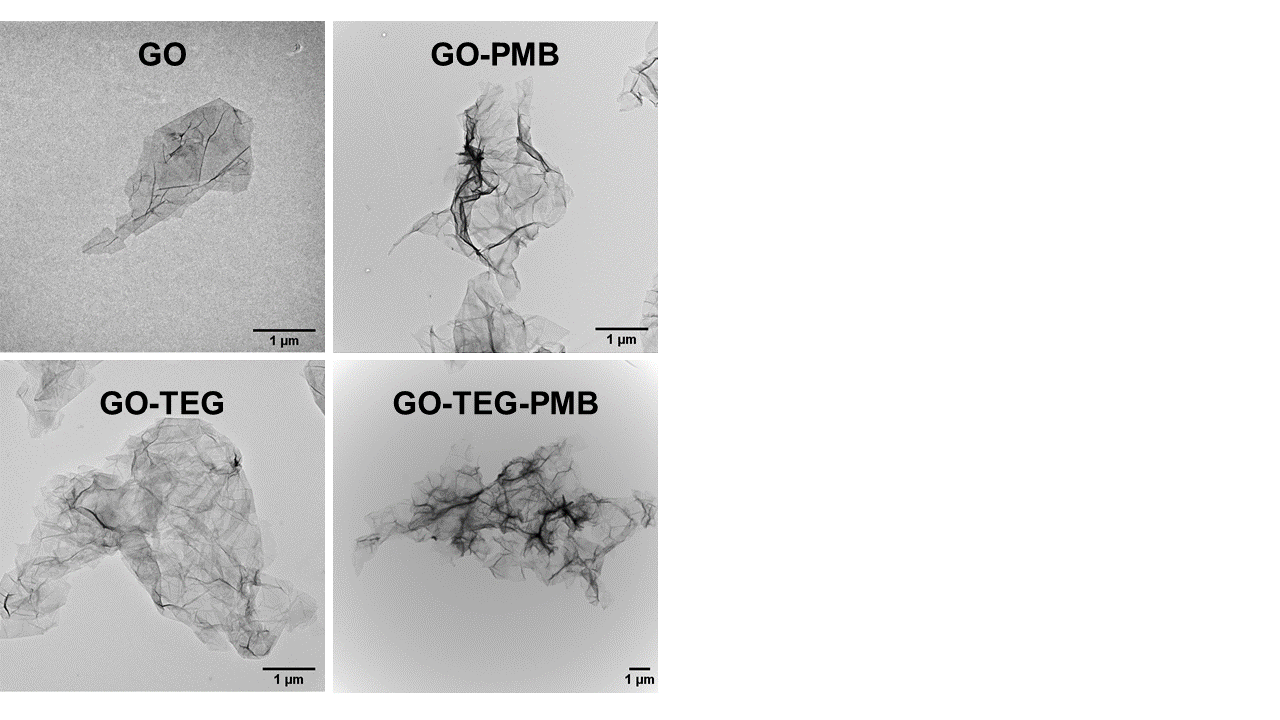
**

**
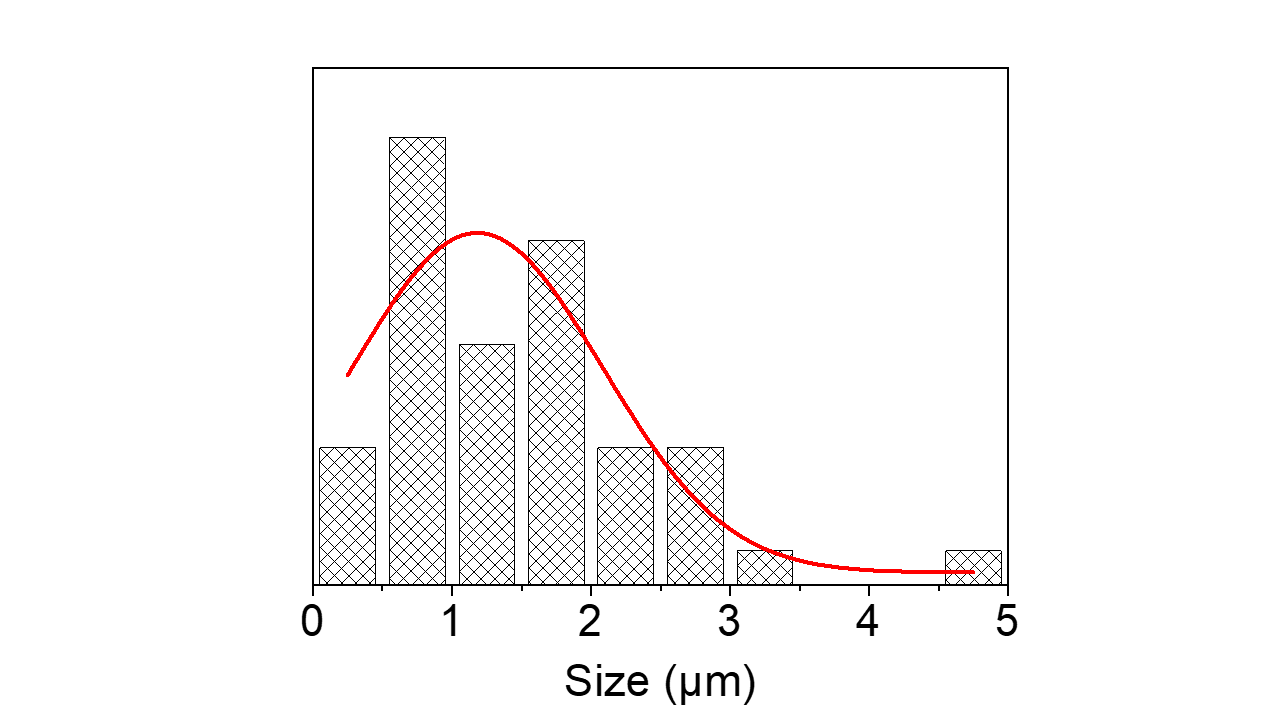
**

**Fig. S1.** TEM images of GO (magnification 30000×), GO-PMB (magnification 25000×), GO-TEG (magnification 25000×) and GO-TEG-PMB (magnification 10000×). Size distribution obtained from TEM images of GO with an average lateral size of 1.49 ± 0.9 µm.


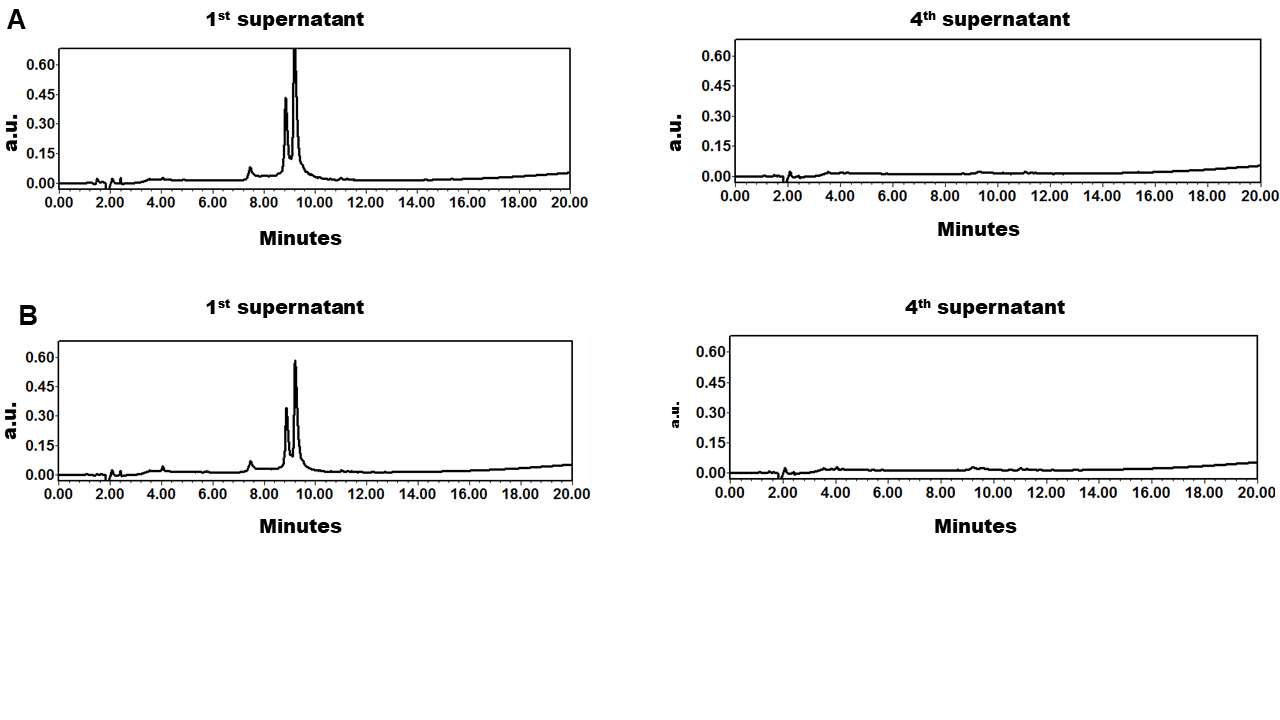


**Fig. S2.** HPLC chromatograms of the 1^st^ supernatant and the 4^th^ supernatant obtained during purification steps of GO-PMB (A) and GO-TEG-PMB (B).

**
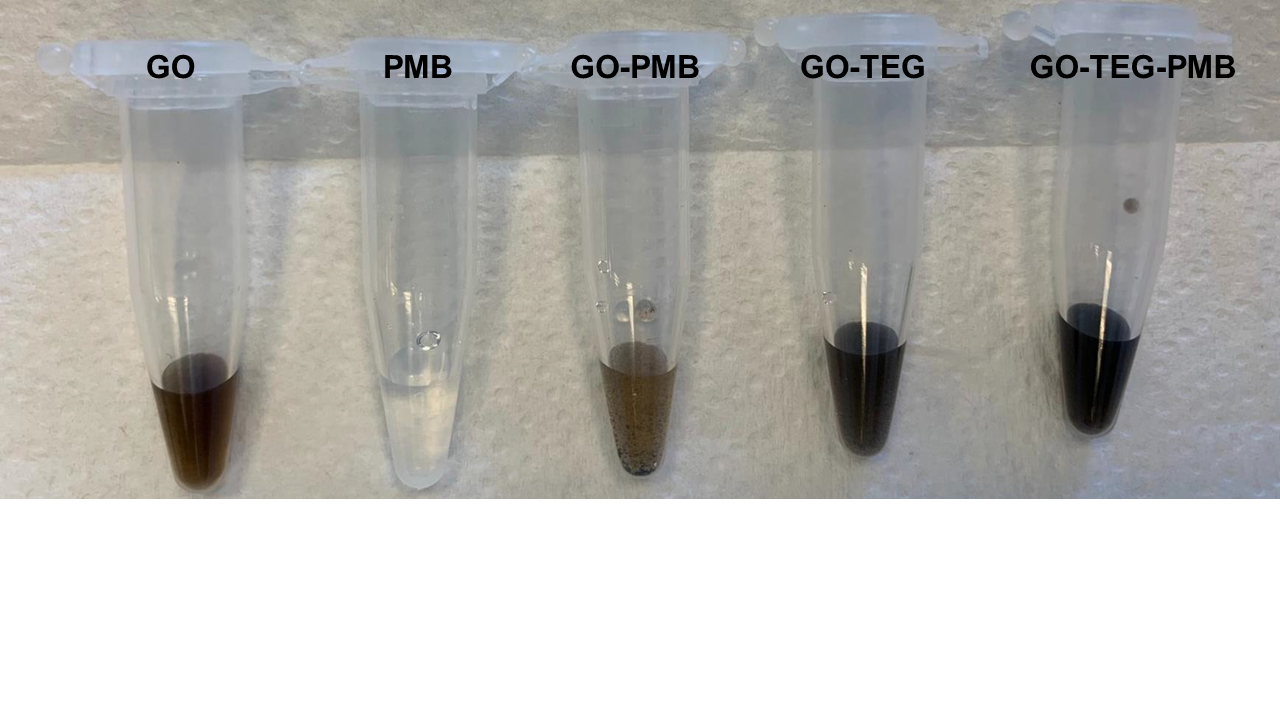
**

**Fig. S3.** Photographs of the different materials in water solution at pH 7 at a concentration of 1 mg/mL.


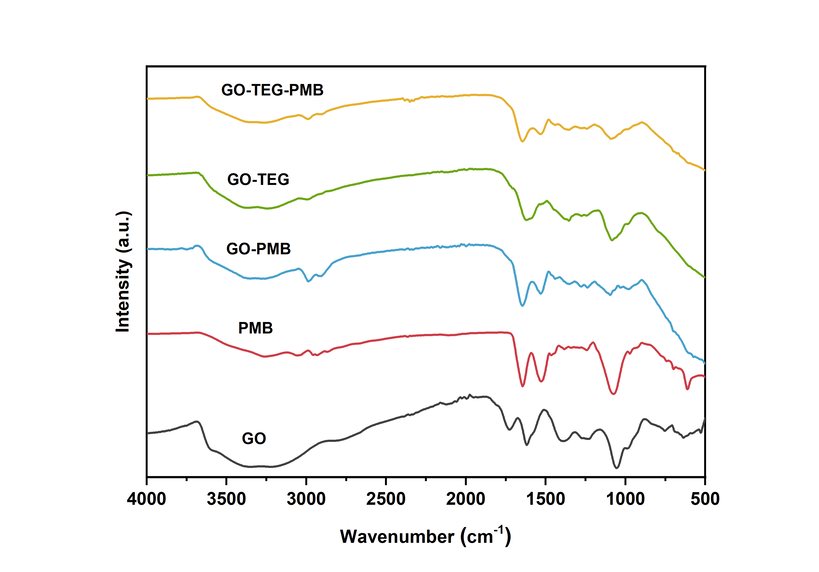


**Fig. S4.** FT-IR spectra of PMB and the different GO materials.
